# Reporting 12 Cases of Maternal Mortality Due to COVID-19; the Role of Termination of Pregnancy as a Double-Edged Sword

**DOI:** 10.34172/aim.2022.121

**Published:** 2022-11-01

**Authors:** Marjan Ghaemi, Sedigheh Hantoushzadeh, Reza Ghanbari, Zohreh Heidary

**Affiliations:** ^1^Vali-e-Asr Reproductive Health Research Center, Tehran University of Medical Sciences, Tehran, Iran; ^2^Gene Therapy Research Center, Digestive Diseases Research Institute, Shariati Hospital, Tehran University of Medical Sciences, Tehran, Iran

## Dear Editor,

 After publishing our last article entitled “maternal and neonatal complications, outcomes and possibility of vertical transmission in Iranian women with COVID-19”^1^ that was published in your valuable journal, we wanted to share our last experience of 12 cases of mortalities due to COVID-19 in pregnant women from January 2021 to July 2021 in a single referral hospital in Iran.

 Pregnant women with COVID-19 are more likely than normal pregnancies to have preterm birth as well as cesarean section. The rate of neonatal intensive care unit (NICU) admission for their neonates is higher, a well.^2^ The results of the previous studies have been contradictory. In some studies, the rates of hospitalization in the intensive care unit (ICU) and mechanical ventilation were higher, although the mortality rate did not increase in comparison with non-pregnant cases. Most women recover without undesirable outcomes, but severe maternal complications as well as maternal and prenatal mortalities have been also reported from COVID-19.^3^

 Here, we report 12 maternal mortalities due to COVID-19 infection in pregnant women. Their vital signs, laboratory data and the delivery mode are listed in Table 1. All women required intensive care unit admission. Totally, 7/12 (58.3%) of the cases were in trimester 3 and the others were in the second trimester. No maternal death before 20 weeks was reported. Most terminations except one were via cesarean section. Half of the women died shortly after pregnancy termination due to fetal or maternal distress. Four newborns remained alive and all were preterm but no history of their COVID-19 test was available. In the other cases where the pregnancy did not terminate, most fetuses were expired due to maternal distress. A severe drop in oxygen saturation and hemoglobin decrease in these individuals can expose the fetus to hypoxia and death.

**Table 1:** Vital Signs, Fetal Status and the Delivery Mode of 12 Maternal Mortalities Due to COVID-19 Infection in Pregnant Women

**Age** **(y)**	**Gravidity**	**O2 sat (%)**	**BP (Sys/Dias)**	**PR**	**Temp**	**RR**	**GA**	**Symptoms & Signs**	**Maternal Terminal Manifestation**	**GA in Termination**	**Termination type**	**Cause of Termination**	**Apgar (5-10 min) or Fetal Status**
26	G2P1	89	110/80	120	38.3	24	34	Fever, cough, myalgia and dyspnea	ARDS	34	C/S	Fetal distress	7
38	G3P1Ab1	98	100/60	98	39	24	25	Fever, chills, myalgia and coughs	ARDS	—	—	—	—
39	G2P1	88	97/60	111	39.5	40	22	Nausea, dyspnea	ARDS	—	—	—	—
35	G6Ab5	90	119/77	140	38.3	24	31	Fever, cough, dyspnea	ARDS, hematuria	32	Vaginal misoprostol	IUFD	IUFD
30	G2P1	92	100/76	100	38.5	35	29	dyspnea	ARDS	29	C/S	fetal distress	1
32	G2P1	60	108/68	120	39.5	40	34	Dyspnea	emphysema	35	C/S	fetal distress	—
28	G2P1	78	124/86	120	39	24	26	Dyspnea	Cardiac arrhythmia	—	—	—	IUFD
29	G3P2	95	110/65	90	37.9	18	35	Cough, dyspnea, decreased smell and taste	ARDS	35	C/S	Uterine contraction	6
21	G1	92	160/110	98	38	22	21	Cough, dyspnea	ARDS, Proteinuria-DIC	—	—	—	IUFD
22	G1	95	100/60	86	37.5	20	32	Cough, dyspnea	ARDS	32	C/S	Fetal distress	7
31	G2p1	80	100/60	128	39.1	40	35	Drowsiness	ARDS	35	C/S	Fetal distress	2-9

O2 sat, O2 saturation; PR, Pulse rate; RR, Respiratory rate; Temp, Temperature; GA, Gestational age; IUFD, Intra uterine fetal death; CS: Cesarean section; BS, Blood sugar; Cr, Creatinine; Hb, Hemoglobin; Plt, Platelet; Sys/Dias, Systolic/diastolic; G, Gravid; P, Para; Ab, Abortion; ARDS, Acute respiratory distress syndrome; DIC, Disseminated intravascular coagulation; IVIG, Intravenous immunoglobulin; WBC, White blood cells; Nut/lymph, Neutrophil to lymphocyte ration; ESR, Erythrocyte sedimentation rate; CRP, C-reactive protein *All vital signs are from their first visit in the hospital.

 On the other hand, the effect of pregnancy on the general condition of mothers is also important. The Royal College of Obstetricians and Gynecologists recommended the mode of delivery to be primarily determined by the obstetric symptoms and mentions that mothers with COVID-19 and their infants should be separated.^4^ Other evidence suggests that cytokine storm is responsible for severe symptoms and mortality in patients with COVID-19 rather than the virus itself. Therefore, cytokine storms during delivery seem to worsen the condition of the mothers. It seems that delivery is like a double-edged sword for mothers. On the one hand, it worsens the condition of mothers, and on the other hand, the risk of fetal distress and demise would be inevitable.
